# Psychotherapy Research During the COVID‐19 Pandemic: Trends in Treatment Modalities and Digital Therapy Uptake (2019–2023)

**DOI:** 10.1002/cpp.70311

**Published:** 2026-07-28

**Authors:** Ian Sivel, Winfried Rief, Cornelia Weise

**Affiliations:** ^1^ Division of Clinical Psychology and Psychotherapy, Department of Psychology Philipps‐University of Marburg Marburg Germany; ^2^ Division of Clinical Psychology and Behavioral Health Technology, Department of Psychology Friedrich‐Alexander‐Universität Erlangen‐Nürnberg Erlangen Germany

**Keywords:** CBT, COVID‐19 pandemic, eHealth, internet‐based interventions, psychotherapy research, randomized controlled trials (RCT), teletherapy, trends

## Abstract

The COVID‐19 pandemic necessitated rapid transitions to digital psychotherapy delivery formats, yet systematic quantification of these developments within psychotherapy research remains limited. This study presents an updated trend analysis of psychotherapy randomized controlled trials (RCTs) from 2019 to 2023. Systematic searches in PubMed, Web of Science and PsycINFO identified RCTs across predefined psychotherapeutic intervention categories. Trials were categorized by treatment approach, region and age group. A total of 1223 psychotherapy RCTs were identified. Despite a temporary decline in 2022 (−13%), the overall number of trials increased by 26.3% across the study period. eHealth interventions constituted the largest psychotherapeutic intervention category (43%), surpassing cognitive behavioural therapy (CBT; 37%). Among eHealth trials, 71% evaluated internet‐delivered CBT. Only eHealth and telehealth showed statistically significant increases in proportional representation over time. Other therapeutic approaches each represented < 7% of treatment arms and showed no significant proportional change. Trial activity remained concentrated in high‐income countries (73.4%) and predominantly targeted adult populations, with children and adolescents comprising 17.3% of trials. The findings illustrate a dynamic field that remained active during the COVID‐19 crisis, with eHealth emerging as the most frequently investigated therapeutic approach accompanied by substantial growth in telehealth interventions. Despite the overall growth in trial activity, research efforts remained heavily concentrated around CBT‐derived interventions, adult populations and high‐income country settings.

## Summary

- The field of psychological treatment research is dynamic and adapting to challenges like the COVID‐19 crisis.
- During the COVID‐19 pandemic, the amount of evaluated eHealth interventions increased disproportionately.
- CBT continues to be the most frequently investigated treatment approach, both in the face‐to‐face and in the eHealth format.
- Traditional therapies such as psychodynamic and systemic approaches maintained a consistently small share without notable change over time (0.8%–2.0% of RCTs), whereas third wave approaches like mindfulness‐based therapies and ACT are investigated more frequently (2.6%–6.9%).
- Psychotherapy research provides practitioners with a continuously increasing robustness of evidence‐based treatment recommendations.

## 1 Introduction

The COVID‐19 pandemic imposed the most profound operational disruption to psychotherapy research in modern history. Its impact extended beyond clinical care, directly affecting how psychological interventions could be evaluated and trialled. Lockdowns and facility closures suspended in‐person studies, disrupted recruitment pipelines and delayed review processes (Lorenc et al. 2023; Sathian et al. 2020; Villarosa et al. 2021). As a result, most non‐COVID clinical research involving human participants was paused, delayed or cancelled (Audisio et al. 2022). Early reports (e.g., APA 2020; Holmes et al. 2020) highlighted the widespread disruption to psychological research workflows during the pandemic and called for rapid methodological adaptation. This prompted regulatory authorities, ethics committees and institutional boards to issue new guidelines, reassess ongoing studies and restructure protocols to accommodate the conditions imposed by the COVID‐19 pandemic (Joshi et al. 2021; Perez et al. 2020). Psychotherapy trials, which often require repeated face‐to‐face contact, faced unique implementation barriers. These were not isolated methodological setbacks but field‐wide constraints that reshaped the conditions under which empirical science could be continued. Understanding how research activity evolved under these conditions is critical, not merely because disruption occurred but because it challenged a research landscape that had remained structurally stable for decades.

Prior to the pandemic, psychotherapy research exhibited notable structural continuity in both trial output and treatment focus. A bibliometric analysis by Soares et al. (2020), covering 28,594 peer‐reviewed articles published between 1970 and 2019, demonstrated the dominance of cognitive‐behavioural therapy (CBT) and a concurrent decline in the representation of psychoanalytical and systemic approaches. Although mindfulness‐ and acceptance‐based interventions gained some momentum during the 2010s, their absolute publication volume remained comparatively limited. Rief et al. (2022), focusing specifically on randomized controlled trials (RCTs) indexed in Web of Science from 2010 to 2019, identified a 343% increase in overall RCT output. However, this expansion was disproportionately concentrated in CBT and eHealth interventions, which accounted for 68% and 18% of trials, respectively. Emerging modalities such as acceptance and commitment therapy (ACT) and mindfulness‐based therapies (MBT) showed only modest gains. Furthermore, trial activity was geographically skewed, with high‐income Anglophone countries dominating the research landscape, whereas contributions from low‐ and middle‐income regions remained marginal. These patterns collectively depict a trial landscape that, despite quantitative growth, remained structurally stable and concentrated within a narrow set of therapeutic paradigms.

The COVID‐19 pandemic introduced an exogenous shock to this previously stable research system of psychotherapy. Whereas prior shifts were shaped by theoretical advances or funding priorities, the COVID‐19 pandemic imposed acute logistical constraints, redefining what could be studied, how and under what conditions. The clinical response to COVID‐19 was rapid. By mid‐2020, over 80% of US psychologists had transitioned to teletherapy (APA 2020), and international surveys documented parallel shifts across diverse health systems (Kinoshita et al. 2022). Although clinical practice adapted swiftly, it remains less clear whether research activity exhibited comparable shifts. Digital treatments remained elevated well beyond the crisis period, suggesting that many adaptations were not merely provisional (Bierbooms et al. 2020; Dang et al. 2021; Molfenter et al. 2021). However, despite assumptions that trial activity slowed, adapted or shifted to digital formats, no systematic empirical mapping has clarified how these changes unfolded in the published record. Key questions remain: Did digital interventions replace or supplement in‐person trials? Did certain treatment approaches gain disproportionate attention? And to what extent did research output decline, recover or restructure during the pandemic years? Answering these questions is consequential. Trial activity not only predetermines which interventions enter clinical guidelines (Clark 2018) but also influences funding allocations (Klavans and Boyack 2017) and practitioner training priorities (Beidas and Kendall 2010). In the absence of timely evaluation, strategic decisions may be guided by outdated assumptions, decoupled from the empirical evolution of the field.

Addressing this gap, our specific aim was to systematically quantify psychotherapy RCT activity between 2019 and 2023. This study provides an empirical basis for understanding how psychotherapy research responded to pandemic‐related constraints, with particular attention to shifts in delivery formats and research focus. Our scope is deliberately narrow: We included only RCTs and a predefined set of treatment families to align with prior mapping approaches (Rief et al. 2022), not to generalize about all research activity but to analyse the field's most influential and high‐quality activities. Our analysis therefore serves as an index of current trends in the published RCT landscape, rather than a comprehensive account of all developments in psychotherapy research. Although former such analyses were based exclusively on Web of Science referenced articles (Rief et al. 2022), we will extend our search to PubMed and PsycINFO. Based on prior trends and pandemic‐induced clinical adaptations, we expected observable shifts in trial activity, particularly in the relative distribution of face‐to‐face versus remote treatment approaches. Accordingly, we pursued exploratory analyses to map treatment approach distributions, demographic targets, regional trends and temporal deviations from prior publication trajectories. By analysing how published psychotherapy trial activity evolved between 2019 and 2023, we aimed to generate insights to inform future research planning and decision‐making among researchers, funders and policy stakeholders.

## 2 Methods

We conducted a structured trend analysis of psychotherapy RCTs, preregistered on AsPredicted (#149876; 6 November 2023). Although this is not a PRISMA‐based systematic review of treatment efficacy, reporting followed relevant PRISMA 2020 elements for database searching, screening, eligibility assessment and study flow documentation (Page et al. 2021). We abstained from primary outcome variables syntheses, meta‐analysis or risk‐of‐bias assessments because the study was designed as a descriptive trend analysis allowing for a focused mapping of short‐term trends in psychotherapy research rather than a full systematic review of treatment efficacy or a bibliometric analysis. Initial hypotheses regarding trial volume trends, treatment modality representation and demographic distributions were preregistered. However, given the unprecedented empirical disruptions associated with the COVID‐19 pandemic, the analytic framework was broadened to include exploratory analyses focused on identifying the most meaningful patterns emerging from the data, aiming to inform future research efforts, whereas the criteria for study inclusion (RCTs) were not changed.

### 2.1 Search Procedure

We collaboratively developed a dual‐string Boolean search strategy and pilot‐tested to optimize precision and recall. One string targeted study design (restricted to title fields per CONSORT recommendations; Schulz et al. 2010), whereas the other indexed treatment approach terms (applied to titles and abstracts, depending on database capabilities). We made adjustments for database‐specific features and systems (e.g., using specific Web of Science categories). Only English‐ and German‐language publications were included. To improve coverage beyond the Web of Science referenced manuscripts (WoS; used by Rief et al. 2022), we included PsycINFO and PubMed to capture clinically relevant trials often underrepresented in WoS. Searches were conducted between November 2023 and May 2024. The full search terms are listed in Table S5.

### 2.2 Eligibility Criteria

Studies were eligible if they met the following criteria:

- Study design: randomized clinical trials (RCTs).
- Psychotherapeutic treatment approach: RCTs had to investigate at least one psychological treatment approach, including cognitive behaviour therapy (CBT), psychodynamic treatments (PDT, including psychoanalysis/analytical psychotherapy and mentalization‐based therapy), internet‐based psychological treatments and other digital approaches using new technologies (eHealth, telehealth), mindfulness‐based therapy (MBT), acceptance and commitment therapy (ACT), interpersonal therapy (IPT), systemic therapy, schema therapy, emotion‐focused therapy (EFT), dialectical behaviour therapy (DBT), cognitive behavioural analysis system of psychotherapy (CBASP) and client‐centered therapy (CCT).
- Publication period: published between January 2019 and December 2023.
- Sample size: RCTs with at least one treatment arm comprising a minimum of 20 participants were included. This threshold was chosen as a practical compromise between ensuring a comprehensive overview of psychotherapy RCTs and setting a minimum study size to support meaningful inclusion in the review.
- Participants: diagnosed mental disorder per DSM‐5 or ICD‐10, including subclinical/high‐risk profiles; trials on chronic pain or tinnitus were included due to recognized psychological comorbidity (Bhatt et al. 2016; Landmark et al. 2024; Stobik et al. 2005), as well as studies involving caregivers if outcomes reflected their therapeutic engagement (Dippel et al. 2022; Weisz et al. 2017).

The treatment approaches were selected to capture major psychotherapeutic intervention categories relevant to research on mental disorders, while avoiding an overly granular taxonomy in which each specific manualized treatment or hybrid intervention would constitute a separate category. Digital and remote categories, including eHealth and telehealth, were included because they were central to the research question and particularly relevant in the context of pandemic‐period psychotherapy research. The classification built on the previous psychotherapy trend analysis by Rief et al. (2022), to preserve conceptual continuity with the earlier work. This was extended by adding further clinically established and research‐relevant intervention categories that had become apparent in the previous work and related screening experience.

Exclusion criteria: prevention‐only studies, secondary analyses (e.g., follow‐up studies if preresults–postresults were already published), protocols, meta‐analyses, corrections, commentaries or studies with included participants with primary medical conditions.

### 2.3 Selection and Classification Process

The first author led a systematic two‐stage selection process: (1) Records that appeared to meet the inclusion criteria during the title‐abstract screening were added to the literature management program, and (2) a full‐text review was conducted to ensure only those meeting all inclusion criteria were retained. Where full‐text access was unavailable, corresponding authors were contacted. Recognizing the risk of classification bias, we implemented a structured quality assurance procedure as a core quality control measure. The first 50 studies were jointly reviewed with supervisors (the two coauthors) to establish a standardized categorization logic, following the structure used by Rief et al. (2022). This calibration phase resulted in a shared coding framework and let to further shaping inclusion criteria and categorization as outlined above. During the main screening, all studies with unclear eligibility or classification were referred to a three‐person consensus group to ensure consistent, nonidiosyncratic decision‐making. When trials were reported in multiple publications (e.g., presenting results at different time points or reporting secondary outcomes), only the publication detailing the primary posttreatment outcomes was considered. Third‐wave interventions (e.g., ACT, MBT and schema therapy) were coded independently from CBT, following Rief et al. (2022), to track their discrete trajectories. For interventions that integrate multiple therapeutic frameworks (e.g., cognitive analytic therapy), we adopted the classification of transtheoretical psychological therapy, as defined by Lutz and Rief (2024). IMIs were classified using a hierarchical approach based on their format and therapeutic model: (1) We distinguished between eHealth interventions (asynchronous and self‐guided formats) and teletherapy (synchronous and therapist‐guided sessions), and (2) for eHealth interventions, the underlying therapeutic orientation was additionally coded where identifiable (e.g., iCBT, iACT, and iPDT).

### 2.4 Analyses

Eligible studies were extracted into Excel, recording study design, psychotherapeutic intervention category, sample size, publication year, country of origin (first author affiliation) and age group. To avoid double‐counting, we maintained two datasets: (1) a trial‐level dataset counting each RCT once to capture overall trends and age distributions and (2) an arm‐level dataset recording each treatment arm separately for each therapeutic intervention category investigated (e.g., a study examining both CBT and ACT was counted twice, once under each therapy), ensuring an accurate representation of therapeutic approaches. We performed descriptive statistical analyses to summarize distributions by psychotherapeutic intervention category, region and age group. To assess deviations from prior publication trends, we projected expected RCT counts by extending the baseline trajectory observed before the 2022 publication decline. Variability was modelled via Monte Carlo simulation (10,000 iterations; Fishman 1996) with normally distributed noise (*σ* = 15) estimated from baseline residuals. We derived empirical 95% prediction intervals from the simulations and assessed whether observed counts fell outside these intervals. Given the short baseline and modelling assumptions, Monte Carlo projections were treated as exploratory descriptive analyses. Pairwise two‐proportion *z* tests with Holm–Bonferroni correction (Agresti 2018; Holm 1979) compared proportional representation of intervention categories between 2019–2021 and 2022–2023 using raw aggregated arm counts. This division was selected post hoc to approximate expected publication lag, as trials affected by the 2020 COVID‐19 onset were more likely to appear from 2022 onward. Tests were restricted to intervention categories with expected cell counts ≥ 5 trials. Cohen's *h* was used to quantify the magnitude of proportional differences between periods (Cohen 1988). For age‐group distributions, risk ratios and binominal test were employed. We conducted all statistical analyses and generated visualizations using Python (Matplotlib).

## 3 Results

We present only the main findings here; comprehensive tables with all relative frequencies are provided in the Supporting Information.

### 3.1 Study Selection

**FIGURE 1:**
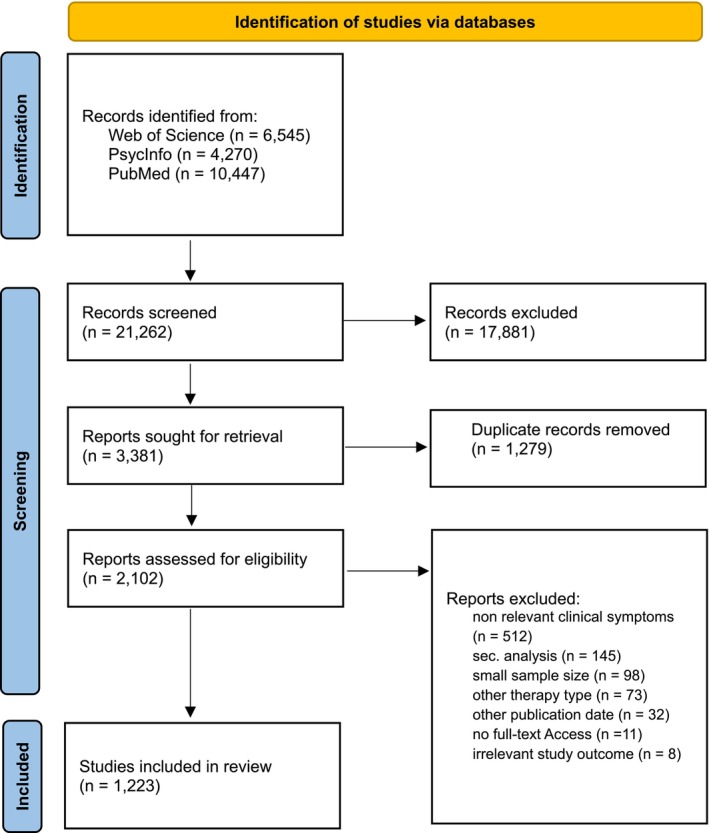
PRISMA flow diagram for RCT selection process.

The systematic search across Web of Science, PsycINFO and PubMed yielded 21,262 hits (see Table S4). After title and abstract screening, 17,881 records were excluded, and 3381 reports were sought for retrieval. After removing 1279 duplicates (37.8%), 2102 reports underwent full‐text screening. Of these, 879 studies were excluded based on the following criteria: absence of (sub)clinical symptoms per ICD‐10/DSM‐5 (58.2%), small sample size (16.5%), secondary analyses (11.1%), other therapy type (8.3%), other publication date (3.6%), no full‐text access (1.3%) and no relevant clinical outcomes (0.9%). The final dataset included 1223 psychotherapy‐related RCTs published between 2019 and 2023 (Figure 1).

### 3.2 Psychotherapy RCT Trends

**FIGURE 2:**
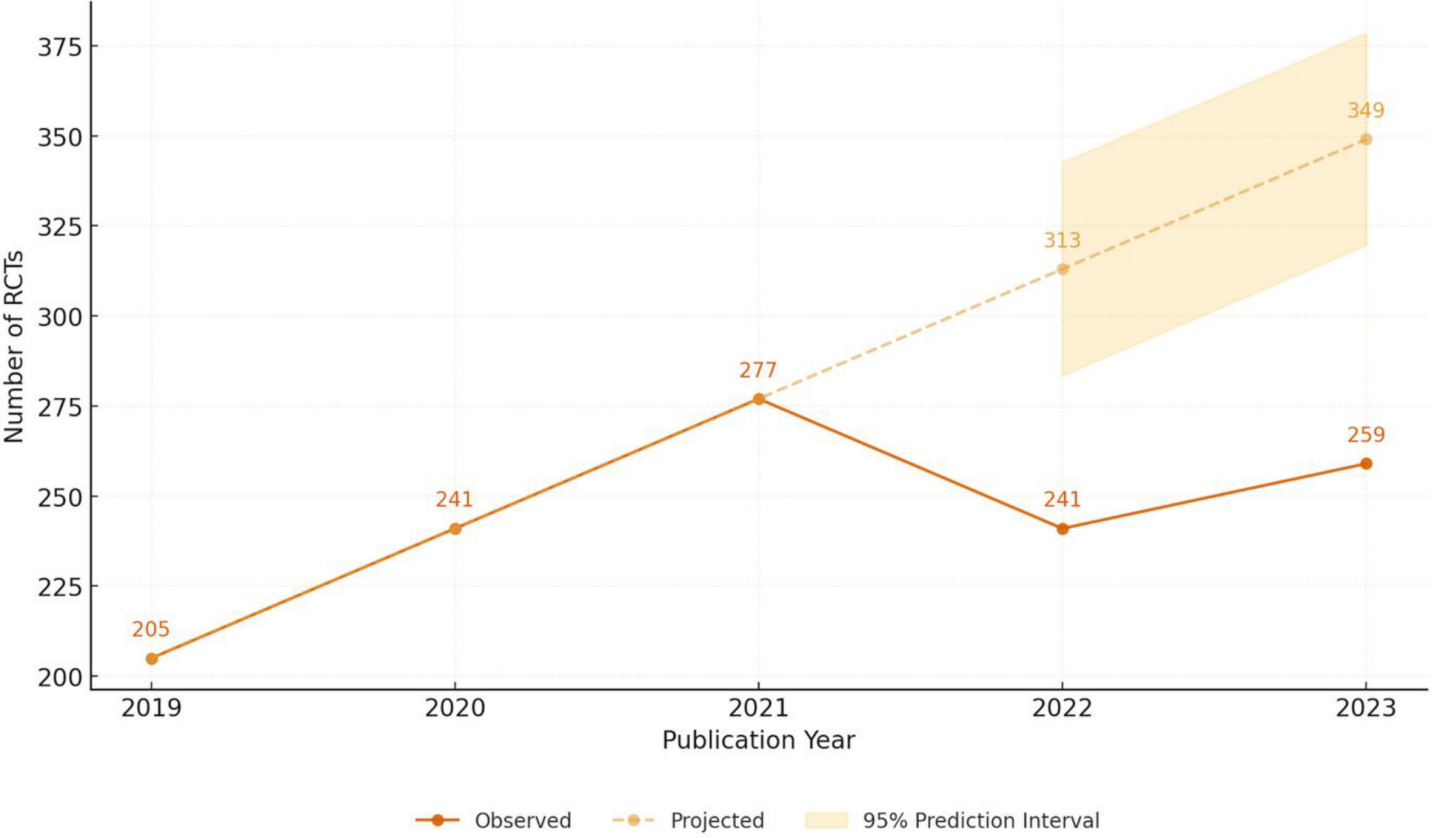
Observed annual RCT publication counts with modelled projections for 2022–2023 *Note*: Solid line indicates the observed number of psychotherapy RCTs published annually from 2019 to 2023. Dashed line represents model‐based projections derived from a linear trend modelled over the 2019–2021 baseline period (+35 trials/year). Shaded area shows the 95% prediction interval for 2022 and 2023, generated via Monte Carlo simulation (10,000 iterations) assuming normally distributed noise (*σ* = 15), with *σ* estimated from residuals of the baseline fit. As the model is based on a short baseline and fixed slope, results are descriptive and exploratory, and should not be interpreted as confirmatory evidence of structural deviation.

The number of RCTs increased by approximately 35%, from 205 in 2019 to 277 in 2021, consistent with the growth trend documented by Rief et al. (2022), as shown in the Supporting Information (Figure S1 and Tables S1 and S2). This trajectory reversed in 2022, which marked a substantial decline (241 trials; −13.0% relative to 2021), followed by a partial rebound in 2023 (259 trials; +7.5% relative to 2022; Figure 2). Based on projections derived from Monte Carlo simulations of growth trends observed pre‐2021, the observed count in 2022 (241 RCTs) fell below the simulation‐derived 95% prediction interval [283.5, 342.6], corresponding to an empirical *p* < 0.001. Similarly, the 2023 count (259 RCTs) fell below the 95% interval [319.7, 378.5], also yielding *p* < 0.001. These results indicate that the observed downturn in RCT publication counts is unlikely to reflect random variation alone. Rather, the deviation from projected trends may plausibly reflect broader systemic influences on clinical trial operations, including but not limited to pandemic‐related disruptions. Between 2019 and 2023, annual psychotherapy RCT output increased overall by approximately 26.3%.

### 3.3 Psychotherapeutic Treatment Category Distributions and Temporal Shifts

Of the 1324 treatment arms identified across psychotherapy RCTs, two modalities dominated the landscape: eHealth (41.8%) and CBT (38.2%), jointly accounting for nearly 80% of all trial activity. All other intervention categories remained below 7% of the total sample (see details Tables S1–S4 and Figures S2 and S3). To assess shifts in empirical focus over time, we compared treatment distributions across two analytic windows (2019–2021: *n* = 778; 2022–2023: *n* = 546). A redistribution of empirical focus was evident: Whereas CBT led during 2019–2021 (42.9%) and eHealth followed (37.7%), this hierarchy reversed in 2022–2023, with eHealth rising to 47.8% and CBT declining to 31.5% (Table 1). Inferential analyses supported these trends: CBT representation decreased significantly (*p* < 0.001, *h* = 0.24), whereas eHealth and Telehealth proportions increased significantly (both *p* < 0.001, *h* = 0.21). In contrast, no significant changes were observed for MBT or ACT (all *p*s > 0.01). Although observed effect sizes were small by conventional benchmarks (Cohen 1988), the consistent pattern of a declining CBT share alongside rising eHealth and Telehealth representation suggests modest but systematic shifts in empirical focus. Given the high share of CBT and eHealth in the psychotherapy RCT literature, even proportional changes of this magnitude imply meaningful reallocations of trial activity toward digitally delivered interventions. When combining eHealth and Telehealth, digitally delivered interventions grew from 40.1% to 54.6% of all treatment arms across periods, marking a substantial shift in delivery format emphasis.

**TABLE 1:** Changes in psychotherapeutic intervention representation between 2019–2021 and 2022–2023.

Category	Annualized treatment‐arm count (2019–2021)	Annualized treatment‐arm count (2022–2023)	Δ (*pp*)	Z	*p* (adj.)	Cohen's *h*
CBT	111.3	86.0	−11.4	4.21	< 0.001*	0.237
MBT	18.7	18.0	−0.6	0.43	0.410	0.024
ACT	7.3	6.0	−0.6	0.71	0.213	0.040
eHealth	97.7	130.5	+10.1	−3.68	< 0.001*	0.205
Telehealth	6.3	18.5	+4.4	−3.68	< 0.001*	0.213

*Note:* Annualized counts represent the number of treatment arms per year within each period (2019–2021 = 3 years; 2022–2023 = 2 years) and are reported solely for descriptive purposes to facilitate interpretation across unequal time spans. All inferential statistics (*Z* values, Holm‐Bonferroni–adjusted *p* values and Cohen's *h* effect sizes) were calculated using raw aggregated counts. *p* values reflect two‐tailed pairwise two‐proportion *z* tests comparing proportional representation between 2019–2021 (*n* = 778) and 2022–2023 (*n* = 546). Tests were restricted to comparisons with expected cell frequencies ≥ 5. Holm‐Bonferroni correction (Holm 1979) was applied to control for multiple comparisons. Asterisk (*) indicates statistical significance after adjustment. Δ (*pp*) denotes the percentage point difference in proportions between periods. Cohen's *h* denotes the effect size for differences in proportions (Cohen 1988), with values of ≥ 0.20, ≥ 0.50 and ≥ 0.80 interpreted as small, medium and large, respectively. Interpretations of temporal trends should consider the unequal duration of observation windows.

Abbreviations: eHealth, asynchronous internet‐based interventions; MBT, mindfulness‐based therapy; Telehealth, synchronous interventions delivered via telecommunication tools.

### 3.4 Psychotherapeutic Orientations Within eHealth Psychotherapy Trials

**FIGURE 3:**
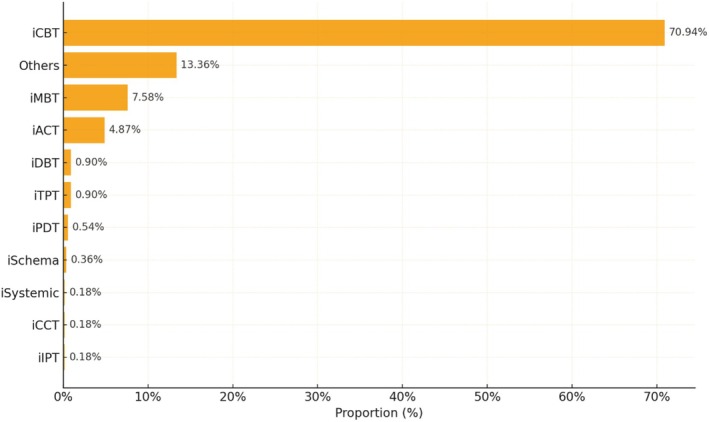
Distribution of therapeutic approaches within eHealth treatment arms (*N* = 554) *Note*: eHealth refers to asynchronous internet‐based interventions and other digital approaches using new technologies. Abbreviations indicate therapeutic approaches delivered in eHealth format: iACT, internet‐based acceptance and commitment therapy; iCBT, internet‐based cognitive behavioural therapy; iCCT, internet‐based client‐centred therapy; iDBT, internet‐based dialectical behaviour therapy; iIPT, internet‐based interpersonal psychotherapy; iMBT, internet‐based mindfulness–based therapy; iPDT, internet‐based psychodynamic therapy; iSchema, internet‐based schema therapy; iSystemic = internet‐based systemic therapy; iTPT, internet‐based transtheoretical therapy. ‘Others’ includes treatment types not fitting clearly into listed categories.

We further examined the composition of eHealth interventions to assess whether digital expansion coincided with increased model diversity. Among the 554 eHealth treatment arms identified, 70.9% were classified as internet‐based CBT (iCBT), with no other orientation exceeding 8% of the digital trial corpus (see Figure 3). The remaining 13.4% were categorized as ‘others’, encompassing innovative or hybridized approaches (e.g., gamified interventions) that could not be clearly aligned with any single established therapeutic orientation.

### 3.5 Regional and Age Group Distribution

**FIGURE 4:**
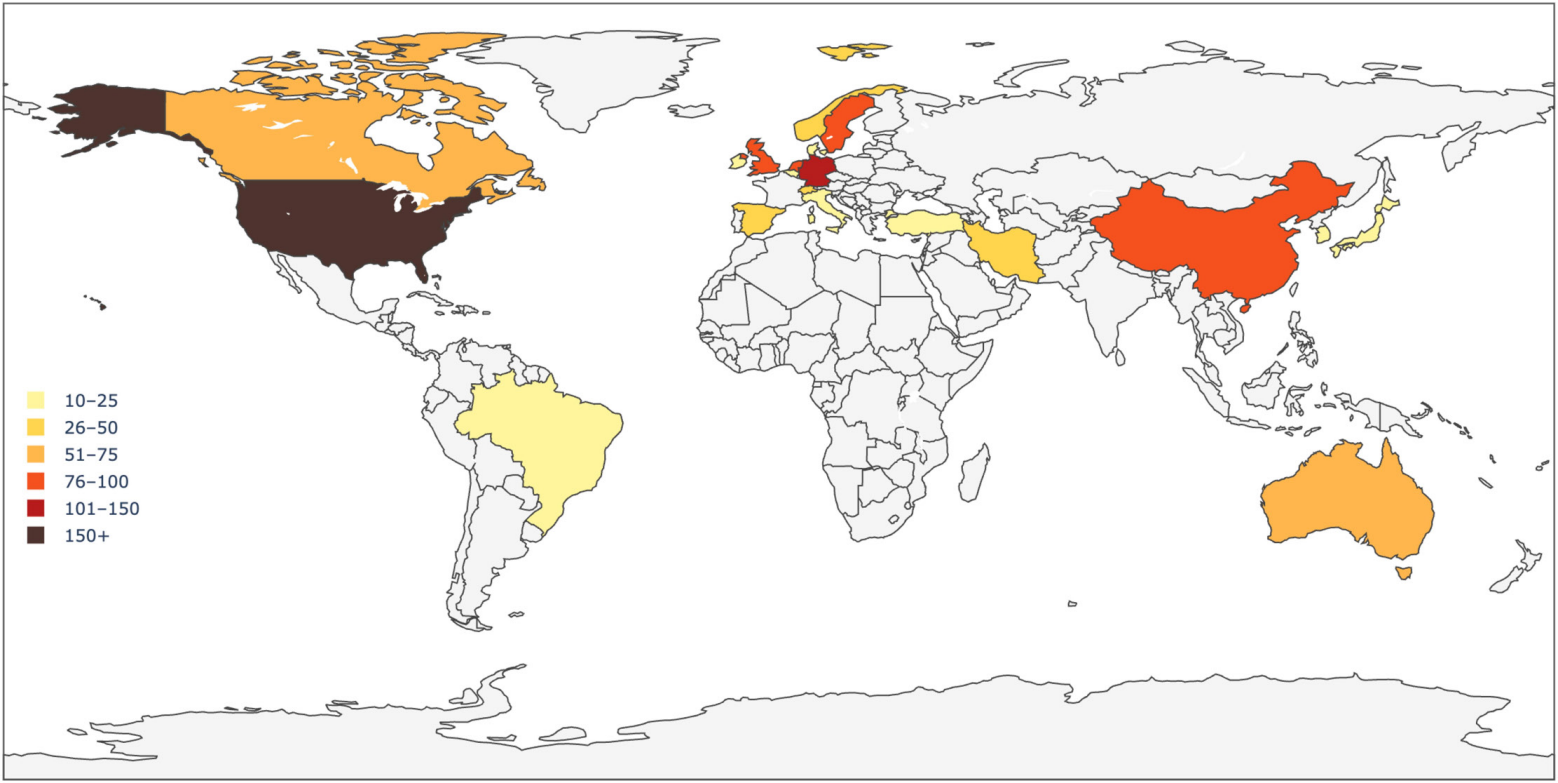
Geographic distribution of treatment arms by country between 2019 and 2023. *Note*: World choropleth map displaying the number of treatment arms per country during the study period 2019–2023, using ordinal colour bins to represent trial volume ranges (e.g., 10–25, 26–50, 51–75, 76–100, 101–150 and 150+). Countries with fewer than 10 treatment arms in total are not displayed.

Trials originated from 52 countries, with research activity concentrated in Europe (41.5%) and North America (31.9%; Figure 4), reflecting a broader concentration in high‐income regions. Regional patterns in trial distribution revealed uneven contributions to specific treatment modalities. Although most countries aligned closely with global averages, several contributed disproportionately high shares to particular intervention types. For instance, China was markedly overrepresented in MBT trials (+149%, 17 arms), whereas the United States showed elevated representation in Telehealth trials (+62%). Sweden (+48%) and Switzerland (+70%) similarly exceeded expected contributions in eHealth research. Norway demonstrated overrepresentation in CBT trials (+54%). Of the 1223 eligible RCTs, 40 (3.3%) mixed‐age trials were excluded, yielding a final analytic sample of 1183 trials. Of these, 978 trials (82.7%) targeted adults and 205 trials (17.3%) targeted children or adolescents. A binomial test demonstrated a significant skew toward adult‐focused trials (*p* < 0.001). The relative likelihood of adult inclusion was 4.77 times higher than that of children/adolescents, with a 95% confidence interval of [4.10, 5.55].

## 4 Discussion

The present study offers an empirical snapshot of how psychotherapy research navigated the unprecedented disruptions imposed by the COVID‐19 pandemic. For decades, the field exhibited uninterrupted growth in RCT activity and sustained theoretical continuity, with CBT consistently anchoring the empirical landscape (Rief et al. 2022; Soares et al. 2020). Our findings reveal that this structural continuity was disrupted during the observed period. RCT activity increased by 26.3% relative to 2019. However, this growth trajectory was nonlinear, with 2022 marking the first recorded decline in trial output in over a decade, followed by a partial recovery in 2023 (see Figure S1; Rief et al. 2022). Concomitant with changes in trial frequency, the distribution of treatment approaches under investigation also shifted. Traditional face‐to‐face delivery formats contracted, whereas digital modalities emerged as the only approaches to sustain consistent growth across the observation window (*p* < 0.001). This transition culminated in a notable reversal in trial representation: eHealth interventions accounted for 43% of all trial activity, modestly surpassing CBT‐focused trials at 37%. This marks a modest shift in the therapeutic model focus of psychotherapeutic research priorities, though not in its theoretical orientation; approximately 71% of eHealth trials iCBT indicates continuity in therapeutic content despite the shift in modality. This pattern likely reflects contextual factors specific to the pandemic period. However, more broadly, these developments should not be viewed solely as pandemic‐driven adaptations but may also reflect increasing societal and health‐system efforts to expand low‐threshold and accessible mental health care, particularly in response to persistent barriers to timely psychological treatment.

Amid reduced clinical access, logistical uncertainty and increased mental health demand (Czeisler et al. 2020; O'Connor et al. 2021; PAHO 2020), protocols that were pre‐existing, manualized and methodologically suited for digital implementation were most readily adopted. iCBT protocols, which had demonstrated feasibility and clinical efficacy prior to the pandemic (Andersson et al. 2016; Carlbring et al. 2018), were particularly well positioned to support continued empirical activity. As such, CBT‐derived interventions, whether delivered digitally or in person, continued to constitute the empirical core of psychotherapy RCT research throughout the observation period. These findings delineate a moment of structural transition in psychotherapy research, wherein logistical adaptation accelerated selective reorganization without fundamentally altering the theoretical architecture of the field.

Beyond the digital–CBT axis, other therapeutic models, including ‘third‐wave’, psychodynamic, systemic and integrative approaches, showed no consistent trajectory, with each representing less than 7% of trial activity throughout the observation window. Although many of these approaches are widely practiced, their limited visibility in the RCT literature challenges their evidence‐based support. Patterns of trial representation carry direct consequences, as they directly shape the inclusion of interventions in clinical guidelines, professional training curricula and implementation systems grounded in evidence‐based policy (APA 2006; Beidas and Kendall 2010). Although the lower trial frequency of some approaches may reflect pandemic‐related constraints, particularly for relational or resource‐intensive models, the overarching pattern is consistent with prior bibliometric evidence of representational asymmetries in the psychotherapy literature (Rief et al. 2022; Soares et al. 2020). These findings provide an updated empirical profile of treatment model representation within the psychotherapy RCT literature and offer a basis for future monitoring of field‐level research priorities.

In addition to the concentration of trial activity around a narrow set of therapeutic models, the findings also reflect persistent imbalances in demographic and geographic representation. Not surprisingly, our findings reflect two well‐documented trends: the predominance of research conducted in high‐income Western countries and a disproportionate focus on adult populations. Populations with the greatest unmet mental health needs, including children, adolescents and individuals in low‐resource settings, remain systemically underrepresented in the very trials used to define what is considered ‘evidence‐based’. Prior analyses have shown that child‐focused research is underrepresented in high‐quality empirical designs such as RCTs (Martinez‐Castaldi et al. 2008), a pattern linked to system‐level barriers including stricter ethics approval processes, complex consent requirements and limited institutional support for youth engagement in research (Hawke et al. 2020; Oliveras et al. 2018; Wadman et al. 2019). Geographical patterns have been linked to a range of structural and logistical constraints: limited funding for research, Western bias in the peer review process, language barriers and restricted global collaboration networks (Amano et al. 2023; Raval et al. 2024). The geographical imbalance is also associated with the predominance of CBT trials. However, other factors besides research interests can influence this effect. CBT approaches may be more easily standardized and exported across contexts, facilitating their investigation and use in addressing global challenges. Selecting exclusively English and German language publications could have further contributed to this asymmetry. These dynamics do not inherently distort the field's knowledge base, but they do warrant vigilance against the risk of overgeneralizing findings derived from demographically and geographically narrow samples.

The pandemic period catalysed significant shifts in psychotherapy research priorities, yet the long‐term consolidation of these developments remains an open question. Our findings indicate an inflection point during the pandemic, wherein digital interventions transitioned from peripheral innovations to central pillars of both clinical delivery and research agendas. This shift aligns with broader transformations in mental health service systems, where remote modalities are increasingly viewed as long‐term infrastructure rather than emergency contingencies (Perrone et al. 2020; van Daele et al. 2021). Still, historical precedents remind us that increased research attention does not always translate into lasting structural integration, as seen with the rise and plateau of mindfulness‐based therapies in the 2010s (Soares et al. 2020). The sustainability of digital modalities will thus depend on strategic ecosystem‐building: deliberate design, institutional endorsement, clinician engagement and policy‐level support. Clinically, IMIs demonstrate substantial promise: ranging from expanded reach and cost‐efficiency to demonstrated symptom improvement across multiple diagnostic categories (Beg and Verma 2024; Domhardt et al. 2021; Mohr et al. 2021; Philippe et al. 2022; van Daele et al. 2021; Wilhelm et al. 2022). Yet the pandemic period uniquely served as a field‐wide stress test, exposing both capacity and fragility. Therapist adoption surged under necessity, but surveys suggest that this exposure also contributed to attitudinal shifts: Many clinicians now express cautious openness toward digital formats, particularly when paired with professional validation and adequate infrastructure (Békés and Aafjes‐van Doorn 2020; Hanley 2021; Rettinger et al. 2023; von Wirth et al. 2024). Blended care models were especially favoured, often preferred over purely digital interventions (Phillips et al. 2022). Concerns about relational shallowness are increasingly challenged by empirical data, which suggest that therapeutic alliance can be established across synchronous and asynchronous digital formats, including among adolescents and young adults (Bucci et al. 2019; Leuchtenberg et al. 2023; Seuling et al. 2024). Beyond accessibility, IMIs offer underexploited scientific opportunities. Digital platforms allow for real‐time personalization, modular experimentation and fine‐grained analysis of therapeutic mechanisms, features with direct implications for theory development and implementation science (Domhardt et al. 2021; Rief et al. 2024). Realizing the potential benefits of digital modalities will require deliberate and sustained strategic investment. Rather than passive continuation of emergency‐driven adaptations, future research should systematically evaluate the clinical efficacy, equity impacts and methodological possibilities afforded by digital platforms. Such efforts will be critical to ensuring that the structural transitions observed during the pandemic evolve into lasting enhancements of both psychotherapeutic science and global mental health care.

### 4.1 Limitations

Several limitations of this study must be acknowledged. First, we focused exclusively on published RCTs. We recognize that other clinically relevant forms of evidence, such as process‐based, qualitative or translationally informed designs, offer valuable insights into therapeutic mechanisms and practice (Hofmann and Hayes 2019). Our focus on published RCTs is probably less indicative of the overall research activities. This is especially true the more the methods of the trials differ from RCTs. We also omitted the search in preregistration databases, and in alternative outlets of research, although publication bias in favour of studies with positive outcomes is still an issue in psychotherapy research (Driessen et al. 2015). Further, a focus on RCTs can amplify differences in research activities between psychotherapeutic approaches, as some approaches are more challenging to translate into an RCT format than others (e.g., long‐term psychodynamic approaches). However, the decision to restrict our scope to RCTs was deliberate: These trials continue to form the basis of national and international treatment guidelines (Clark 2018; Rief et al. 2022). We also excluded RCTs with group sizes below 20. In this case, it cannot be expected that randomization will work and lead to groups that are comparable on all variables except the experimental condition. This focus enables structural insight into the evidence base most directly linked to formal clinical recommendations.

Second, the classification system relied on registry‐assigned intervention categories. This may underrepresent novel, hybrid or culturally embedded approaches that resist standardized labelling and thus limits visibility into conceptual innovation occurring outside dominant taxonomies. However, open science recommendations would encourage and request that even these approaches should be preregistered. Nevertheless, although we included several modern developments (e.g., mentalization‐based treatments were categorized as psychodynamic therapies), it is almost impossible to provide comprehensive coverage of all psychological treatments. For example, therapies such as Emotional Awareness and Expression Therapy (EAET) were not retrieved. In addition, using search terms such as CBT or psychodynamic therapy obscures the fact that these are not homogeneous and constant treatment families, but continuously evolving categories (e.g., consider the incorporation of mindfulness‐, ACT‐ or value‐based approaches into CBT; Rief et al. 2026). Hybrid and emerging approaches can be hidden with our search strategy. Therefore, similar frequencies for CBT can still indicate significant changes in the content being investigated.

Third, the 5‐year observation window provides only a near‐term view of pandemic‐adjacent research activity. Because publication year does not necessarily correspond to trial conduct, the observed temporal patterns should be interpreted as trends in the published record rather than direct indicators. Several developments started already before the COVID‐19 crisis (e.g., increase in eHealth‐ and m‐Health interventions) but further accelerated during this period. Although sufficient to detect emerging patterns, it does not allow prediction of their long‐term trajectory, and rebound effects can occur. We view this study as an early diagnostic signal, one that can inform future, extended analyses of evolving post‐pandemic research dynamics. Finally, we did not assess the methodological quality or reporting standards of included trials. This study aimed to map structural trends in trial activity, not to evaluate trial rigor. Future research could usefully combine structural mapping with quality‐weighted analyses to offer a more differentiated picture of evidence production and uptake.

## 5 Conclusions

Psychotherapy research maintained empirical continuity during the COVID‐19 pandemic by rapidly adjusting delivery formats and procedures. This adaptation centred largely on digital modalities, particularly within CBT‐based protocols. These developments illustrate that the field is not static but continues to respond actively to scientific, technological and global challenges. Whether this reflects pragmatic adaptation or structural inertia remains an open question. What is clear, however, is that psychotherapy research operates within a broader sociopolitical context in which the need for scalable, evidence‐based mental health care is rapidly intensifying. Ongoing empirical observation will be critical to determine whether the adaptations observed during this period reflect enduring structural changes or temporary redistributions driven by extraordinary external constraints. To ensure that such adaptations are guided by robust scientific standards rather than short‐term pragmatism, critical reflection on emerging research trajectories will be essential. Our findings offer an updated empirical profile of treatment model representation within psychotherapy research and a foundation for future monitoring of research priorities at the field level.

## Funding

The authors have nothing to report.

## Conflicts of Interest

The authors declare no conflicts of interest.

## Supporting information




**Figure S1:** Number of included Treatment Arms per year in Rief et al. (2022) and the present study (2010–2023).
**Table S1:** Number of Included Treatment Arms per Year by Psychotherapeutic Intervention in Rief et al. (2022) and the Present Study.
**Table S2:** Regional Distribution of Treatment Arms across Psychotherapeutic Interventions from 2019 to 2023.
**Table S3:** Annaual Growth Rates of Overall RCTs and Treatment Arms by Psychotherapeutic Intervention from 2019 to 2023.
**Figure S2:** Proportions of included Treatment Arms across Psychotherapeutic Interventions between 2019–2023.
**Figure S3:** Annual Absolute Change in Proportions of Treatment Arms in CBT, eHealth and Telehealth.
**Table S4:** Search Hits by Search Category.
**Table S5:** Search Terms in Web of Science, PsycINFO and PubMed.

## Data Availability

The data that support the findings of this study are available on request from the corresponding author.
