# Supplementary material for: Psychotherapy Research During the COVID‐19 Pandemic: Trends in Treatment Modalities and Digital Therapy Uptake (2019–2023)
Source: Clin Psychol Psychother. 2026 Jul 28;33(4):e70311. doi: 10.1002/cpp.70311 (PMC13411721; doi:10.1002/cpp.70311)
Supplement: Supplementary file 1 — Figure S1: Number of included Treatment Arms per year in Rief et al. (2022) and the present study (2010–2023). Table S1: Number of Included Treatment Arms per Year by Psychotherapeutic Intervention in Rief et al. (2022) and the Present Study. Table S2: Regional Distribution of Treatment Arms across Psychotherapeutic Interventions from 2019 to 2023. Table S3: Annaual Growth Rates of Overall RCTs and Treatment Arms by Psychotherapeutic Intervention from 2019 to 2023. Figure S2: Proportions of included Treatment Arms across Psychotherapeutic Interventions between 2019–2023. Figure S3: Annual Absolute Change in Proportions of Treatment Arms in CBT, eHealth and Telehealth. Table S4: Search Hits by Search Category. Table S5: Search Terms in Web of Science, PsycINFO and PubMed. [file CPP-33-e70311-s001.docx]

**Supplementary Material to the article:**Psychotherapy Research during the COVID-19 Pandemic: Trends in Treatment Modalities and Digital Therapy Uptake (2019–2023)

Figure S1

*
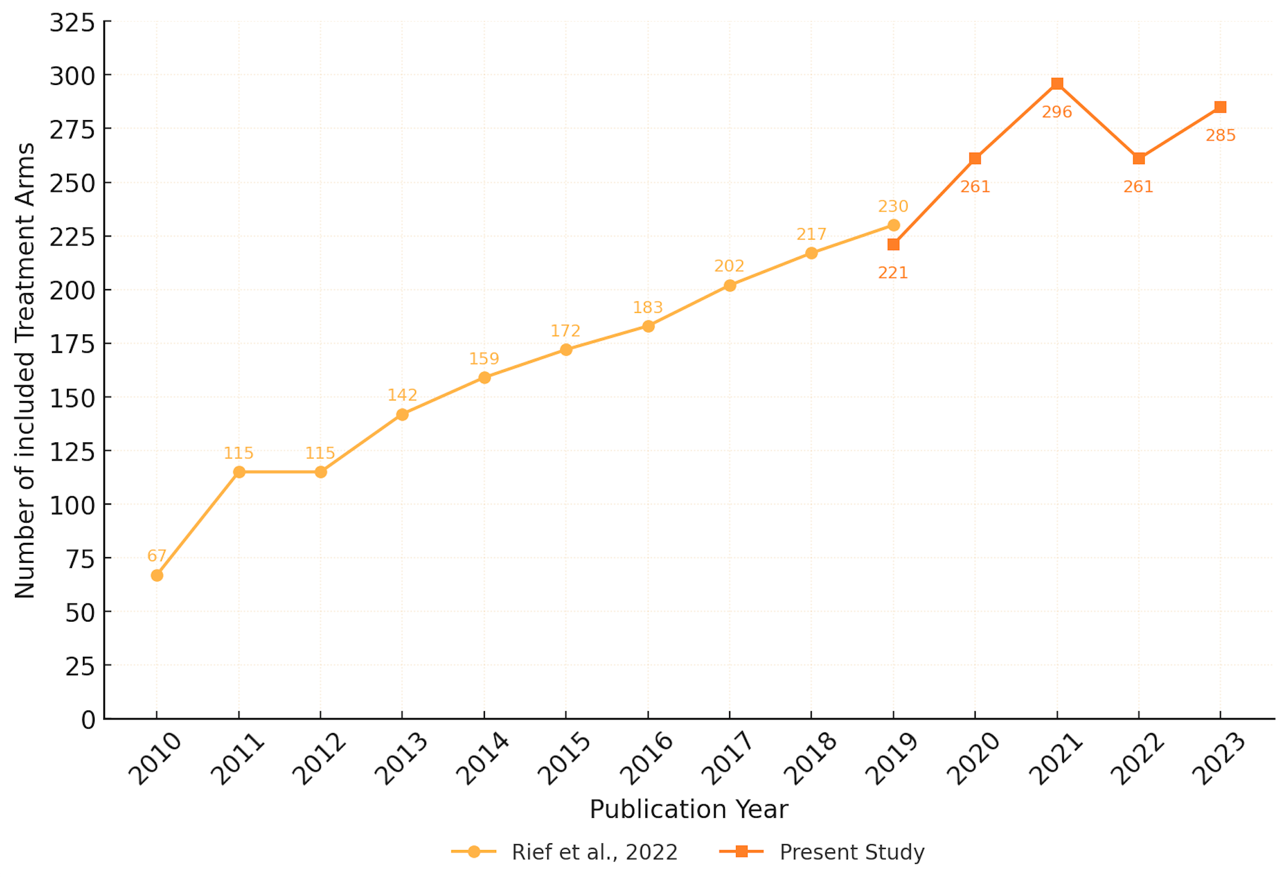
Number of included Treatment Arms per year in Rief et al. (2022) and the present study (2010–2023)*

*Note*. Interpretations of longitudinal comparisons should account for methodological differences between the datasets. Rief et al. (2022) included only studies indexed in Web of Science and focused on a narrower set of treatment modalities. The present study incorporates a broader scope, including trials identified in Web of Science, PubMed, and PsycINFO, and covers an expanded range of therapeutic approaches.

Table S1

*Number of Included Treatment Arms per Year by Psychotherapeutic Intervention in Rief et al. (2022) and the Present Study*

| Study | Publication  Year | CBT | eHealth | MBT | ACT | PDT | Systemic | IPT | Schema | EFT | Telehealth | DBT | CCT | TPT | CBASP |
| --- | --- | --- | --- | --- | --- | --- | --- | --- | --- | --- | --- | --- | --- | --- | --- |
| Rief et al. (2022) | 2010 | 43 | 12 | 1 | 3 | 3 | 2 | 3 | 0 | 0 |  |  |  |  |  |
|  | 2011 | 77 | 21 | 0 | 4 | 2 | 3 | 7 | 0 | 1 |  |  |  |  |  |
|  | 2012 | 72 | 20 | 0 | 7 | 8 | 2 | 5 | 1 | 0 |  |  |  |  |  |
|  | 2013 | 96 | 29 | 1 | 3 | 8 | 1 | 2 | 2 | 0 |  |  |  |  |  |
|  | 2014 | 108 | 31 | 0 | 5 | 7 | 2 | 4 | 1 | 1 |  |  |  |  |  |
|  | 2015 | 126 | 28 | 1 | 6 | 5 | 3 | 3 | 3 | 0 |  |  |  |  |  |
|  | 2016 | 128 | 33 | 3 | 6 | 4 | 2 | 4 | 1 | 2 |  |  |  |  |  |
|  | 2017 | 133 | 42 | 3 | 7 | 9 | 1 | 6 | 0 | 1 |  |  |  |  |  |
|  | 2018 | 154 | 35 | 5 | 11 | 4 | 2 | 3 | 1 | 2 |  |  |  |  |  |
|  | 2019 | 157 | 43 | 7 | 9 | 3 | 3 | 5 | 1 | 2 |  |  |  |  |  |
|  | Total | 1094 | 294 | 21 | 61 | 53 | 21 | 42 | 10 | 9 |  |  |  |  |  |
|  | | | | | | | | | | | | | | | |
| Present Study | 2019 | 103 | 80 | 16 | 5 | 6 | 4 | 1 | 0 | 0 | 3 | 2 | 1 | 0 | 0 |
|  | 2020 | 107 | 99 | 16 | 9 | 8 | 4 | 3 | 1 | 0 | 8 | 4 | 0 | 2 | 0 |
|  | 2021 | 124 | 114 | 24 | 8 | 3 | 2 | 1 | 1 | 2 | 8 | 5 | 3 | 1 | 0 |
|  | 2022 | 90 | 123 | 20 | 5 | 2 | 1 | 2 | 1 | 3 | 11 | 2 | 1 | 0 | 0 |
|  | 2023 | 82 | 138 | 16 | 7 | 8 | 0 | 6 | 0 | 0 | 26 | 1 | 0 | 1 | 0 |
|  | Total | 506 | 554 | 92 | 34 | 27 | 11 | 13 | 3 | 5 | 56 | 14 | 5 | 4 | 0 |

*Note*. Interpretations of longitudinal comparisons should account for methodological differences between the datasets. Rief et al. (2022) included only studies indexed in Web of Science and focused on a narrower set of treatment modalities. The present study incorporates a broader scope, including trials identified in Web of Science, PubMed, and PsycINFO, and covers an expanded range of therapeutic approaches.

Table S2

*Regional Distribution of Treatment Arms across Psychotherapeutic Interventions from 2019 to 2023*

| Country of Orgin | eHealth | CBT | MBT | Telehealth | ACT | PDT | DBT | IPT | Systemic | EFT | CCT | TPT | Schema | Totals |
| --- | --- | --- | --- | --- | --- | --- | --- | --- | --- | --- | --- | --- | --- | --- |
| USA | 132 | 134 | 28 | 23 | 11 | 3 | 2 | 8 | 5 | 1 | 2 | 1 | 0 | 350 |
| Germany | 60 | 49 | 6 | 2 | 0 | 1 | 2 | 0 | 1 | 0 | 0 | 0 | 0 | 121 |
| China | 34 | 39 | 17 | 3 | 1 | 1 | 0 | 1 | 0 | 0 | 0 | 1 | 0 | 97 |
| UK | 40 | 33 | 4 | 1 | 1 | 4 | 2 | 1 | 1 | 0 | 1 | 1 | 0 | 89 |
| Netherlands | 31 | 38 | 8 | 0 | 1 | 3 | 0 | 0 | 0 | 0 | 1 | 0 | 2 | 84 |
| Sweden | 48 | 18 | 0 | 1 | 4 | 3 | 1 | 0 | 1 | 0 | 0 | 0 | 1 | 77 |
| Australia | 37 | 27 | 1 | 3 | 0 | 1 | 1 | 0 | 0 | 0 | 0 | 1 | 0 | 71 |
| Canada | 30 | 19 | 5 | 5 | 1 | 0 | 3 | 0 | 1 | 0 | 0 | 0 | 0 | 64 |
| Spain | 16 | 18 | 6 | 2 | 1 | 0 | 0 | 0 | 0 | 0 | 0 | 0 | 0 | 43 |
| Norway | 6 | 17 | 1 | 0 | 1 | 1 | 1 | 0 | 1 | 1 | 0 | 0 | 0 | 29 |
| Switzerland | 20 | 5 | 0 | 1 | 0 | 0 | 0 | 0 | 1 | 1 | 0 | 0 | 0 | 28 |
| Iran | 7 | 12 | 2 | 3 | 1 | 1 | 1 | 0 | 0 | 1 | 0 | 0 | 0 | 28 |
| Brazil | 3 | 12 | 3 | 0 | 1 | 2 | 0 | 1 | 0 | 0 | 0 | 0 | 0 | 22 |
| Denmark | 8 | 9 | 1 | 0 | 1 | 2 | 0 | 0 | 0 | 0 | 0 | 0 | 0 | 21 |
| Turkey | 8 | 8 | 0 | 0 | 0 | 1 | 0 | 0 | 0 | 0 | 1 | 0 | 0 | 18 |
| Japan | 7 | 7 | 1 | 2 | 0 | 0 | 0 | 0 | 0 | 0 | 0 | 0 | 0 | 17 |
| Italy | 3 | 6 | 1 | 1 | 0 | 0 | 0 | 2 | 0 | 0 | 0 | 0 | 0 | 13 |
| Korea | 8 | 4 | 0 | 0 | 1 | 0 | 0 | 0 | 0 | 0 | 0 | 0 | 0 | 13 |
| Ireland | 6 | 1 | 0 | 1 | 3 | 0 | 0 | 0 | 0 | 1 | 0 | 0 | 0 | 12 |
| Belgium | 3 | 3 | 1 | 1 | 1 | 1 | 0 | 0 | 0 | 0 | 0 | 0 | 0 | 10 |

*Note.* Only countries with a minimum total of 10 treatment arms are displayed. Database: Web of Science, PsycINFO and PubMed. MBT = mindfulness-based therapy.

Table S3

*Annaual Growth Rates of Overall RCTs and Treatment Arms by Psychotherapeutic Intervention from 2019 to 2023*

| Publication  Year | RCTs |  | Treatment Arms (%) | | | | | | | | |  |
| --- | --- | --- | --- | --- | --- | --- | --- | --- | --- | --- | --- | --- |
|  | Overall | | eHealth | CBT | MBT | Telehealth | ACT | PDT | DBT | IPT | Systemic | |
| 2020 | +17.6% | | +23.8% | +3.9% | 0% | +166.7% | +80% | +33.3% | +100% | +200% | 0% | |
| 2021 | +14.9% | | +15.2% | +15.9% | +50% | 0% | -11.1% | -62.5% | +25% | -66.7% | -50% | |
| 2022 | -13% | | +7.9% | -27.4% | -16.7% | +37.5% | -37.5% | -33.3% | -60% | +100% | -50% | |
| 2023 | +7.5% | | +12.2% | -8.9% | -20% | +136.4% | +40% | +300% | -50% | +200% | -100% | |

*Note*. Database: Web of Science, PsycINFO and PubMed. eHealth = asynchronous internet-based therapies and other digital approaches using new technologies; CBT = cognitive behavior therapy; MBT = mindfulness-based therapy; Telehealth = synchronous therapies conducted via telecommunication tools; ACT = acceptance and commitment therapy; PDT = Psychodynamic/-analytical therapy; DBT = dialectical behavior therapy; IPT = interpersonal therapy. Emotion-focused treatments, Client-centered therapy, Transtheoretical psychological therapy and Cognitive behavioral analysis system of psychotherapy were excluded from the growth rate calculation due to insufficient data.

Figure S2

*Proportions of included Treatment Arms across Psychotherapeutic Interventions between 2019-2023*


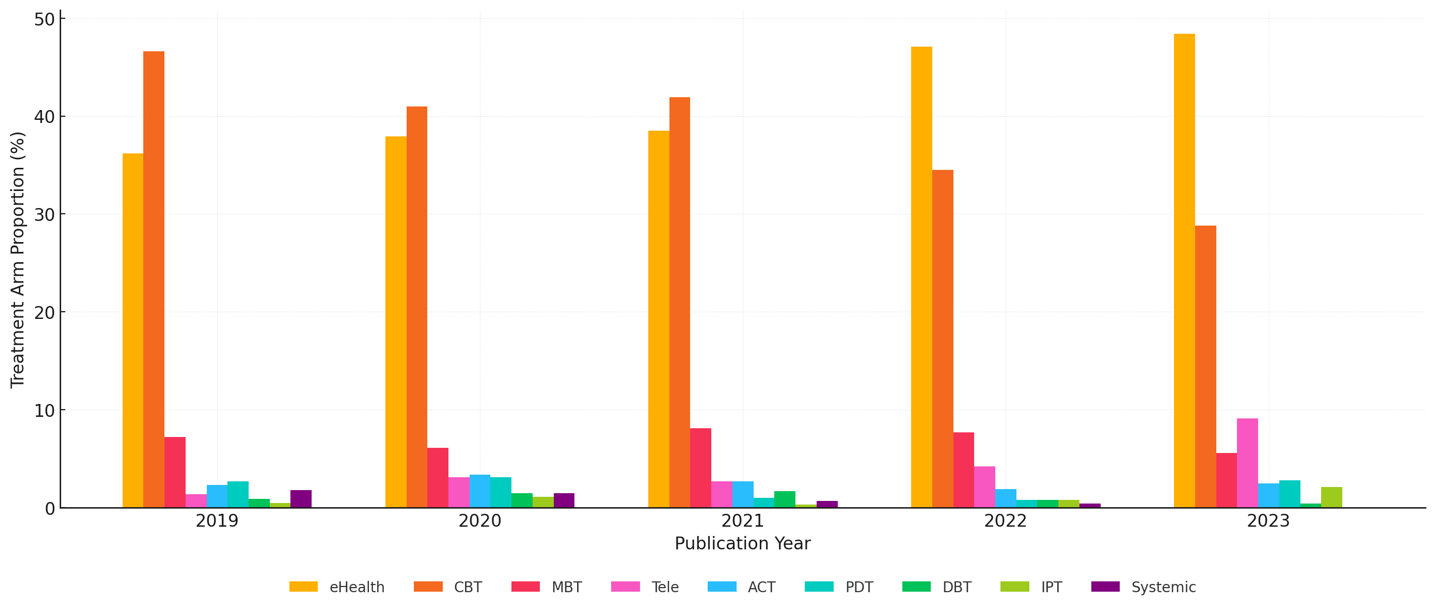


*Note.* eHealth = asynchronous internet-based therapies and other digital approaches using new technologies; CBT = cognitive behavior therapy; MBT = mindfulness-based therapy; Telehealth = synchronous therapies conducted via telecommunication tools; ACT = acceptance and commitment therapy; PDT = psychodynamic/-analytical therapy; DBT = dialectical behavior therapy; IPT = interpersonal therapy. Treatment approaches with fewer than 10 treatment arms in total were excluded.

Figure S3
*Annual Absolute Change in Proportions of Treatment Arms in CBT, eHealth and Telehealth*


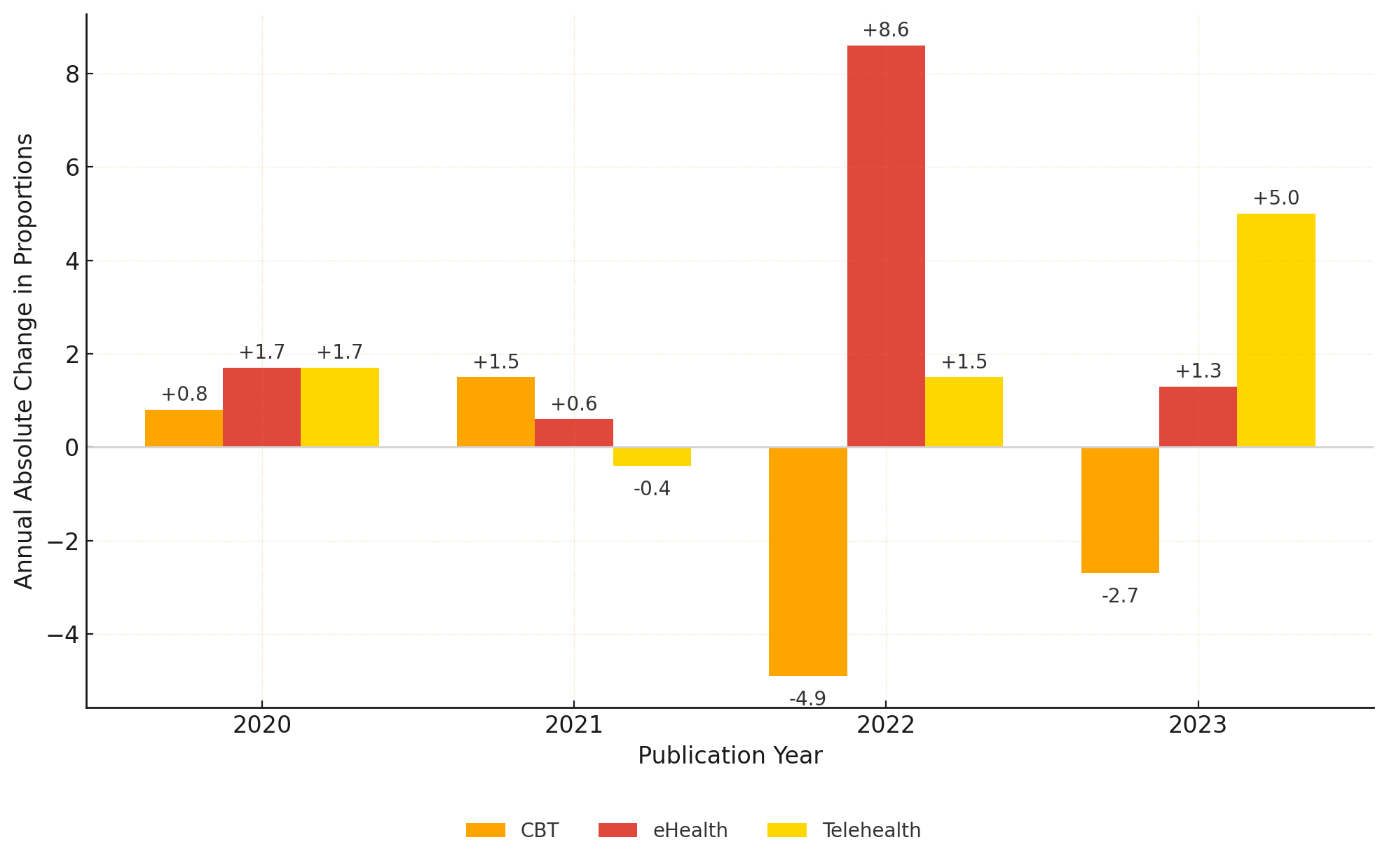
*Note*. Bars represent the absolute year-over-year differences in the proportion of treatment arms, expressed in percentage points (*pp*). Positive values indicate increased representation from the previous year; negative values indicate declines. Other categories showed minimal fluctuations across all years (≤ ±1.5*pp*) and were excluded for visual clarity.

Table S4
*Search Hits by Search Category*

| Search Category | WoS Hits | PsychInfo Hits | PubMed Hits | Total Hits |
| --- | --- | --- | --- | --- |
| EFT | 48 | 37 | 59 | 144 |
| IPT | 45 | 37 | 60 | 142 |
| Schema | 15 | 17 | 30 | 62 |
| CBASB | 1 | 1 | 1 | 3 |
| DBT | 45 | 34 | 61 | 140 |
| PDT | 81 | 83 | 111 | 275 |
| CCT | 165 | 67 | 525 | 757 |
| Systemic | 105 | 79 | 343 | 527 |
| MBT | 807 | 586 | 963 | 2356 |
| ACT | 597 | 268 | 639 | 1504 |
| CBT | 2586 | 1879 | 3090 | 7555 |
| IMIs | 2050 | 1182 | 4565 | 7797 |
| Total | 6545 | 4270 | 10447 | 21262 |

*Note.* Search results from Web of Science, PsycINFO and PubMed. Abbreviations: EFT = emotion-focused therapy; IPT = interpersonal psychotherapy; CBASB = cognitive behavioral analysis system of psychotherapy; DBT = dialectical behavior therapy; PDT = psychodynamic/-analytical therapy; CCT = client-centered therapy; MBT = mindfulness-based therapy; ACT = acceptance and commitment therapy, CBT = cognitive behavioral therapy, IMIs = internet- and mobile based interventions. IMIs includes both eHealth and telehealth interventions.

Table S5

*Search Terms in Web of Science, PsycINFO and PubMed*

| Emotion-focused Therapy | | Web of Science |  |  |
| --- | --- | --- | --- | --- |
| Search formula | AND | TI = (RCT OR randomi*) NOT TI = (protocol OR "meta analysis" OR review OR “secondary analysis” OR "non randomi*") |  |  |
|  |  | AB = (EFT OR EFCT OR EFT-C OR "emotion* focused" OR "emotion* based" OR "emotion* oriented") |  |  |
| Publication Years | | 01/2019 and 12/2023 |  |  |
| Document Types | | Articles |  |  |
| Languages | | English, German |  |  |
| Web of Science Categories | | • Psychiatry  • Psychology Clinical  • Integrative Complementary Medicine  • Clinical Neurology  • Psychology Developmental  • Neurosciences  • Public Environmental Occupational   Health  • Family Studies  • Health Care Sciences Services  • Multidisciplinary Sciences  • Psychology Multidisciplinary  • Psychology Biological  • Social Sciences Biomedical |  |  |
| Interpersonal Therapy | | Web of Science |  |  |
| Search formula | AND | TI = (RCT OR randomi*) NOT TI = (protocol OR "meta analysis" OR review OR “secondary analysis” OR "non randomi*") |  |  |
|  |  | AB = (IPT OR “interpersonal therapy” OR “interpersonal psychotherapy” OR “interpersonal psychological” OR "interpersonal treatment" OR "interpersonal intervention") |  |  |
| Publication Years | | 01/2019 and 12/2023 |  |  |
| Document Types | | Articles |  |  |
| Languages | | English, German |  |  |
| Web of Science Categories | | • Psychiatry  • Psychology Clinical  • Psychology  • Clinical Neurology  • Psychology Developmental  • Public Environmental Occupational   Health  • Health Care Sciences Services  • Psychology Multidisciplinary  • Psychology Psychoanalysis  • Rehabilitation  • Social Sciences Interdisciplinary  • Multidisciplinary Sciences  • Health Policy Services |  |  |
| Schema Therapy | | Web of Science |  |  |
| Search formula | AND | TI = (RCT OR randomi*) NOT TI = (protocol OR "meta analysis" OR review OR “secondary analysis” OR "non randomi*") |  |  |
|  |  | AB = (schema) |  |  |
| Publication Years | | 01/2019 and 12/2023 |  |  |
| Document Types | | Articles |  |  |
| Languages | | English, German |  |  |
| Web of Science Categories | | • Psychiatry  • Psychology Clinical  • Health Care Sciences Services  • Psychology  • Psychology Developmental  • Psychology Educational  • Public Environmental Occupational   Health |  |  |
| Cognitive Behavioral Analysis System of Psychotherapy | | Web of Science |  |  |
| Search formula | AND | TI = (RCT OR randomi*) NOT TI = (protocol OR "meta analysis" OR review OR “secondary analysis” OR "non randomi*") |  |  |
|  |  | AB = ("Cognitive Behavioral Analysis System of Psychotherapy" OR CBASP) |  |  |
| Publication Years | | 01/2019 and 12/2023 |  |  |
| Document Types | | Articles |  |  |
| Languages | | English, German |  |  |
| Web of Science Categories | | - Psychology Clinical |  |  |
| Dialectical Behavior Therapy | | Web of Science |  |  |
| Search formula | AND | TI = (RCT OR randomi*) NOT TI = (protocol OR "meta analysis" OR review OR “secondary analysis” OR "non randomi*") |  |  |
|  |  | AB = (DBT* OR dialecti*) |  |  |
| Publication Years | | 01/2019 and 12/2023 |  |  |
| Document Types | | Articles |  |  |
| Languages | | English, German |  |  |
| Web of Science Categories | | • Psychiatry  • Psychology Clinical  • Clinical Neurology  • Psychology  • Psychology Developmental  • Health Care Sciences Services  • Neurosciences  • Behavioral Sciences  • Psychology Educational  • Psychology Multidisciplinary  • Rehabilitation  • Psychology Social |  |  |
| Psychodynamic Therapy | | Web of Science |  |  |
| Search formula | AND | TI = (RCT OR randomi*) NOT TI = (protocol OR "meta analysis" OR review OR “secondary analysis” OR "non randomi*") |  |  |
|  |  | AB = (psychodynami* OR stpp OR psychoanaly* OR transference OR mentali* OR “supportive expressive" OR "analytic therapy" OR “analytic psychotherapy” OR “analytic treatment” OR “dynamic therapy” OR “dynamic psychotherapy” OR “dynamic treatment” OR “dynamic intervention” ) |  |  |
| Publication Years | | 01/2019 and 12/2023 |  |  |
| Document Types | | Articles |  |  |
| Languages | | English, German |  |  |
| Web of Science Categories | | • Psychiatry  • Psychology Clinical  • Psychology  • Psychology Developmental  • Clinical Neurology  • Psychology Multidisciplinary  • Public Environmental Occupational   Health  • Pediatrics  • Health Care Sciences Services  • Multidisciplinary Sciences  • Family Studies  • Neurosciences  • Psychology Applied  • Psychology Educational  • Psychology Psychoanalysis  • Psychology Social  • Rehabilitation  • Substance Abuse |  |  |
| Client-centered Therapy | | Web of Science |  |  |
| Search formula | AND | TI = (RCT OR randomi*) NOT TI = (protocol OR "meta analysis" OR review OR “secondary analysis” OR "non randomi*") |  |  |
|  |  | AB = (“process experiential” OR "person center*" OR “patient center*” OR "client center*" OR "non directive" OR Rogerian OR humanistic OR “person focus*” OR “client focus” OR “patient focus*” OR “person oriented” OR “client oriented” OR “patient oriented”) |  |  |
| Publication Years | | 01/2019 and 12/2023 |  |  |
| Document Types | | Articles |  |  |
| Languages | | English, German |  |  |
| Web of Science Categories | | • Health Care Sciences Services  • Public Environmental Occupational   Health  • Clinical Neurology  • Psychiatry  • Pediatrics  • Psychology Clinical  • Rehabilitation  • Multidisciplinary Sciences  • Neurosciences  • Primary Health Care  • Education Educational Research  • Social Sciences Interdisciplinary  • Health Policy Services  • Psychology Developmental  • Family Studies  • Social Sciences Biomedical  • Substance Abuse  • Psychology Educational |  |  |
| Systemic Therapy | | Web of Science |  |  |
| Search formula | AND | TI = (RCT OR randomi*) NOT TI = (protocol OR "meta analysis" OR review OR “secondary analysis” OR "non randomi*") |  |  |
|  |  | AB = ("systemic therap*” OR “system oriented” OR “family therap*” OR “family based” OR “family oriented” OR "systemic family therapy" OR "Systemic Couple Therapy" OR "Systemic Group Therapy" OR "Systemic Approach*" OR "Systemic Intervention*" OR "Systemic Techni*" OR multisystemic ) |  |  |
| Publication Years | | 01/2019 and 12/2023 |  |  |
| Document Types | | Articles |  |  |
| Languages | | English, German |  |  |
| Web of Science Categories | | • Psychiatry  • Pediatrics  • Psychology Clinical  • Public Environmental Occupational   Health  • Psychology Developmental  • Family Studies  • Health Care Sciences Services  • Clinical Neurology  • Psychology  • Health Policy Services  • Rehabilitation  • Social Sciences Biomedical  • Psychology Multidisciplinary  • Multidisciplinary Sciences  • Behavioral Sciences  • Substance Abuse  • Psychology Social  • Psychology Educational |  |  |
| Mindfullness-based Therapy | | Web of Science |  |  |
| Search formula | AND | TI = (RCT OR randomi*) NOT TI = (protocol OR "meta analysis" OR review OR “secondary analysis” OR "non randomi*") |  |  |
|  |  | AB = (mindful* OR MBCT OR MBSR OR MBEAT ) |  |  |
| Publication Years | | 01/2019 and 12/2023 |  |  |
| Document Types | | Articles |  |  |
| Languages | | English, German |  |  |
| Web of Science Categories | | • Psychiatry  • Psychology Clinical  • Health Care Sciences Services  • Psychology Multidisciplinary  • Clinical Neurology  • Neurosciences  • Public Environmental Occupational   Health  • Psychology  • Multidisciplinary Sciences  • Integrative Complementary Medicine  • Psychology Developmental  • Rehabilitation  • Psychology Applied  • Substance Abuse  • Education Educational Research  • Health Policy Services  • Behavioral Sciences  • Pediatrics  • Psychology Educational  • Social Sciences Interdisciplinary  • Psychology Social  • Social Sciences Biomedical  • Psychology Experimental  • Family Studies  • Primary Health Care  • Development Studies  • Psychology Biological  • Psychology Psychoanalysis |  |  |
| Acceptance and Commitment Therapy | | Web of Science |  |  |
| Search formula | AND | TI = (RCT OR randomi*) NOT TI = (protocol OR "meta analysis" OR review OR “secondary analysis” OR "non randomi*") |  |  |
|  |  | AB = ("psychological flexibility" OR ACT OR "acceptance and commitment" OR "acceptance commitment" |  |  |
| Publication Years | | 01/2019 and 12/2023 |  |  |
| Document Types | | Articles |  |  |
| Languages | | English, German |  |  |
| Web of Science Categories | | • Psychiatry  • Psychology Clinical  • Integrative Complementary Medicine  • Clinical Neurology  • Psychology Developmental  • Neurosciences  • Public Environmental Occupational   Health  • Family Studies  • Health Care Sciences Services  • Multidisciplinary Sciences  • Psychology Multidisciplinary  • Psychology Biological  • Social Sciences Biomedical |  |  |
| Cognitive Behavioural Therapy | | Web of Science |  |  |
| Search formula | AND | TI = (RCT OR randomi*) NOT TI = (protocol OR "meta analysis" OR review OR “secondary analysis” OR "non randomi*") |  |  |
|  |  | AB = (CBT OR GCBT OR "process based" OR "trauma focus*" OR "TF-CBT" OR "cognitive based" OR "cognitive therap*" OR "cognitive treatment*" OR "cognitive intervention*" OR "cognitive behavio*" OR "cognitive training" OR "meta cognitive" OR metacognitive OR "behavio* activation" OR "behavio* therap*" OR "behavio* treatment*" OR "behavio* intervention*" OR "behavio* training" OR “exposure therap*” OR "emotion* regulation" OR "social skills training" OR "social skills therapy" OR "social skills treatment" OR "social skills intervention" OR "cognitive restructur*" OR "relaxation training" OR "relaxation therapy" OR "relaxation treatment" OR "relaxation intervention" OR "PMR" OR "autogenic" OR "progressive muscle relaxation" OR "biofeedback" OR "neurofeedback”) |  |  |
| Publication Years | | 01/2019 and 12/2023 |  |  |
| Document Types | | Articles |  |  |
| Languages | | English, German |  |  |
| Web of Science Categories | | • Psychiatry  • Psychology Clinical  • Clinical Neurology  • Neurosciences  • Health Care Sciences Services  • Psychology  • Public Environmental Occupational   Health  • Psychology Multidisciplinary  • Rehabilitation  • Psychology Developmental  • Substance Abuse  • Pediatrics  • Multidisciplinary Sciences  • Behavioral Sciences  • Social Sciences Biomedical  • Family Studies  • Psychology Educational  • Health Policy Services  • Integrative Complementary Medicine  • Psychology Applied  • Psychology Experimental  • Social Sciences Interdisciplinary  • Primary Health Care  • Psychology Social  • Education Educational Research  • Psychology Biological  • Psychology Psychoanalysis |  |  |
| Internet- and Mobile-based Therapy | | Web of Science |  |  |
| Search formula | AND | TI = (RCT OR randomi*) NOT TI = (protocol OR "meta analysis" OR review OR “secondary analysis” OR "non randomi*") |  |  |
|  |  | AB = (eHealth OR “e-Health” OR “electronic Health” OR “mHealth” OR “m-Health” OR “mobile Health” OR “e mental” OR “e therapy” OR “icbt” OR “ccbt” OR “computer assisted” OR “online therap*” OR “online treatment*” OR “online intervention*” OR “online training” OR “ online base*” OR “online delivered” OR “internet therap*” OR “internet treatment*” OR “internet intervention*” OR “internet base*” OR “internet delivered” OR “digital therap*” OR “digital intervention*” OR “digital treatment*” OR “therapy app” OR “mobile intervention” OR “mobile app*” OR “mobile therapy” OR “mobile treatment” OR "web therap*" OR “web based therap*” OR “web based intervention*” OR smartphone OR “online self help” OR “video therapy” OR “virtual therapy” OR “virtual reality” OR “virtual intervention” OR “self guided therapy” OR “self help app” OR “guided self help” OR “blended care” OR "blended treatment" OR "blended therap*" OR "blended intervention" OR “digital mental” OR “app based” OR “telehealth” OR "telemental health" OR "remote therap*" OR "remote care" OR "gamified therap*" OR "gamification") |  |  |
| Publication Years | | 01/2019 and 12/2023 |  |  |
| Document Types | | Articles |  |  |
| Languages | | English, German |  |  |
| Web of Science Categories | | • Health Care Sciences Services  • Psychiatry  • Psychology Clinical  • Clinical Neurology  • Rehabilitation  • Neurosciences  • Pediatrics  • Psychology  • Psychology Multidisciplinary  • Multidisciplinary Sciences  • Substance Abuse  • Health Policy Services  • Education Educational Research  • Psychology Developmental  • Social Sciences Biomedical  • Primary Health Care  • Psychology Applied  • Behavioral Sciences  • Social Sciences Interdisciplinary  • Psychology Social  • Family Studies  • Psychology Educational  • Psychology Experimental  • Psychology Biological  • Psychology Psychoanalysis |  |  |
| Emotion-focused Therapy | | APA PsycInfo |  |  |
| Search formula | AND | TI = (RCT OR randomi*) NOT TI = (protocol OR "meta analysis" OR review OR “secondary analysis” OR "non randomi*") |  |  |
|  |  | AB = (EFT OR EFCT OR EFT-C OR "emotion* focused" OR "emotion* based" OR "emotion* oriented") |  |  |
| Publication Years | | 01/2019 and 12/2023 |  |  |
| Expanders | | Apply equivalent subjects |  |  |
| Languages | | English, German |  |  |
| Search modes | | Proximity |  |  |
| Interpersonal Therapy | | APA PsycInfo |  |  |
| Search formula | AND | TI = (RCT OR randomi*) NOT TI = (protocol OR "meta analysis" OR review OR “secondary analysis” OR "non randomi*") |  |  |
|  |  | AB = (IPT OR “interpersonal therapy” OR “interpersonal psychotherapy” OR “interpersonal psychological” OR "interpersonal treatment" OR "interpersonal intervention") |  |  |
| Publication Years | | 01/2019 and 12/2023 |  |  |
| Expanders | | Apply equivalent subjects |  |  |
| Languages | | English, German |  |  |
| Search modes | | Proximity |  |  |
| Schema Therapy | | APA PsycInfo |  |  |
| Search formula | AND | TI = (RCT OR randomi*) NOT TI = (protocol OR "meta analysis" OR review OR “secondary analysis” OR "non randomi*") |  |  |
|  |  | AB = schema |  |  |
| Publication Years | | 01/2019 and 12/2023 |  |  |
| Expanders | | Apply equivalent subjects |  |  |
| Languages | | English, German |  |  |
| Search modes | | Proximity |  |  |
| Cognitive Behavioral Analysis System of Psychotherapy | | APA PsycInfo |  |  |
| Search formula | AND | TI = (RCT OR randomi*) NOT TI = (protocol OR "meta analysis" OR review OR “secondary analysis” OR "non randomi*") |  |  |
|  |  | AB = ("Cognitive Behavioral Analysis System of Psychotherapy" OR CBASP) |  |  |
| Publication Years | | 01/2019 and 12/2023 |  |  |
| Expanders | | Apply equivalent subjects |  |  |
| Languages | | English, German |  |  |
| Search modes | | Proximity |  |  |
| Dialectical Behavior Therapy | | APA PsycInfo |  |  |
| Search formula | AND | TI = (RCT OR randomi*) NOT TI = (protocol OR "meta analysis" OR review OR “secondary analysis” OR "non randomi*") |  |  |
|  |  | AB = (DBT OR dialecti*) |  |  |
| Publication Years | | 01/2019 and 12/2023 |  |  |
| Expanders | | Apply equivalent subjects |  |  |
| Languages | | English, German |  |  |
| Search modes | | Proximity |  |  |
| Psychodynamic Therapy | | APA PsycInfo |  |  |
| Search formula | AND | TI = (RCT OR randomi*) NOT TI = (protocol OR "meta analysis" OR review OR “secondary analysis” OR "non randomi*") |  |  |
|  |  | AB = (psychodynami* OR stpp OR psychoanaly* OR transference OR mentali* OR “supportive expressive" OR "analytic therapy" OR “analytic psychotherapy” OR “analytic treatment” OR “dynamic therapy” OR “dynamic psychotherapy” OR “dynamic treatment” OR “dynamic intervention”) |  |  |
| Publication Years | | 01/2019 and 12/2023 |  |  |
| Expanders | | Apply equivalent subjects |  |  |
| Languages | | English, German |  |  |
| Search modes | | Proximity |  |  |
| Client-centered Therapy | | APA PsycInfo |  |  |
| Search formula | AND | TI = (RCT OR randomi*) NOT TI = (protocol OR "meta analysis" OR review OR “secondary analysis” OR "non randomi*") |  |  |
|  |  | AB = ("process experiential” OR "person center*" OR “patient center*” OR "client center*" OR "non directive" OR Rogerian OR humanistic OR “person focus*” OR “client focus” OR “patient focus*” OR “person oriented” OR “client oriented” OR “patient oriented”) |  |  |
| Publication Years | | 01/2019 and 12/2023 |  |  |
| Expanders | | Apply equivalent subjects |  |  |
| Languages | | English, German |  |  |
| Search modes | | Proximity |  |  |
| Systemic Therapy | | APA PsycInfo |  |  |
| Search formula | AND | TI = (RCT OR randomi*) NOT TI = (protocol OR "meta analysis" OR review OR “secondary analysis” OR "non randomi*") |  |  |
|  |  | AB = ("systemic therap*” OR “system oriented” OR “family therap*” OR “family based” OR “family oriented” OR "systemic family therapy" OR "Systemic Couple Therapy" OR "Systemic Group Therapy" OR "Systemic Approach*" OR "Systemic Intervention*" OR "Systemic Techni*" OR multisystemic) |  |  |
| Publication Years | | 01/2019 and 12/2023 |  |  |
| Expanders | | Apply equivalent subjects |  |  |
| Languages | | English, German |  |  |
| Search modes | | Proximity |  |  |
| Mindfulness-based Therapy | | APA PsycInfo |  |  |
| Search formula | AND | TI = (RCT OR randomi*) NOT TI = (protocol OR "meta analysis" OR review OR “secondary analysis” OR "non randomi*") |  |  |
|  |  | AB = ( mindful* OR MBCT OR MBSR OR MBEAT ) |  |  |
| Publication Years | | 01/2019 and 12/2023 |  |  |
| Expanders | | Apply equivalent subjects |  |  |
| Languages | | English, German |  |  |
| Search modes | | Proximity |  |  |
| Acceptance and Commitment Therapy | | APA PsycInfo |  |  |
| Search formula | AND | TI = (RCT OR randomi*) NOT TI = (protocol OR "meta analysis" OR review OR “secondary analysis” OR "non randomi*") |  |  |
|  |  | AB = ("psychological flexibility" OR act OR "acceptance and commitment" OR "acceptance commitment") |  |  |
| Publication Years | | 01/2019 and 12/2023 |  |  |
| Expanders | | Apply equivalent subjects |  |  |
| Languages | | English, German |  |  |
| Search modes | | Proximity |  |  |
| Cognitive Behavioral Therapy | | APA PsycInfo |  |  |
| Search formula | AND | TI = (RCT OR randomi*) NOT TI = (protocol OR "meta analysis" OR review OR “secondary analysis” OR "non randomi*") |  |  |
|  |  | AB = (CBT OR GCBT OR "process based" OR "trauma focus*" OR "TF-CBT" OR "cognitive based" OR "cognitive therap*" OR "cognitive treatment*" OR "cognitive intervention*" OR "cognitive behavio*" OR "cognitive training" OR "meta cognitive" OR metacognitive OR "behavio* activation" OR "behavio* therap*" OR "behavio* treatment*" OR "behavio* intervention*" OR "behavio* training" OR “exposure therap*” OR "emotion* regulation" OR "social skills training" OR "social skills therapy" OR "social skills treatment" OR "social skills intervention" OR "cognitive restructuring" OR "relaxation training" OR "relaxation therapy" OR "relaxation treatment" OR "relaxation intervention" OR "PMR" OR "autogenic" OR "progressive muscle relaxation" OR "biofeedback" OR "neurofeedback”) |  |  |
| Publication Years | | 01/2019 and 12/2023 |  |  |
| Expanders | | Apply equivalent subjects |  |  |
| Languages | | English, German |  |  |
| Search modes | | Proximity |  |  |
| Internet- and Mobile-based Therapy | | APA PsycInfo |  |  |
| Search formula | AND | TI = (RCT OR randomi*) NOT TI = (protocol OR "meta analysis" OR review OR “secondary analysis” OR "non randomi*") |  |  |
|  |  | AB = (eHealth OR “e-Health” OR “electronic Health” OR “mHealth” OR “m-Health” OR “mobile Health” OR “e mental” OR “e therapy” OR “icbt” OR “ccbt” OR “computer assisted” OR “online therap*” OR “online treatment*” OR “online intervention*” OR “online training” OR “ online base*” OR “online delivered” OR “internet therap*” OR “internet treatment*” OR “internet intervention*” OR “internet base*” OR “internet delivered” OR “digital therap*” OR “digital intervention*” OR “digital treatment*” OR “therapy app” OR “mobile intervention” OR “mobile app*” OR “mobile therapy” OR “mobile treatment” OR "web therap*" OR “web based therap*” OR “web based intervention*” OR smartphone OR “online self help” OR “video therapy” OR “virtual therapy” OR “virtual reality” OR “virtual intervention” OR “self guided therapy” OR “self help app” OR “guided self help” OR “blended care” OR "blended treatment" OR "blended therap*" OR "blended intervention" OR “digital mental” OR “app based” OR “telehealth” OR "telemental health" OR "remote therap*" OR "remote care" OR "gamified therap*" OR "gamification") |  |  |
| Publication Years | | 01/2019 and 12/2023 |  |  |
| Expanders | | Apply equivalent subjects |  |  |
| Languages | | English, German |  |  |
| Search modes | | Proximity |  |  |
| Emotion-focused Therapy | | PubMed |  |  |
| Search formula | AND | (("RCT"[Title] OR "randomi*"[Title]) NOT ("protocol"[Title] OR "meta analysis"[Title] OR "review"[Title] OR "secondary analysis"[Title] OR "non randomi*"[Title])) |  |  |
|  |  | ("eft"[Title/Abstract] OR "EFCT"[Title/Abstract] OR "EFT-C"[Title/Abstract] OR "emotion focused"[Title/Abstract] OR "emotional focused"[Title/Abstract] OR "emotionally focused"[Title/Abstract] OR "emotion based"[Title/Abstract] OR "emotional based"[Title/Abstract] OR "emotionally based"[Title/Abstract] OR "emotion oriented"[Title/Abstract] OR "emotional oriented"[Title/Abstract] OR "emotionally oriented"[Title/Abstract]) |  |  |
| Publication Years | | 01/2019 and 12/2023 |  |  |
| Languages | | English, German |  |  |
| Interpersonal Therapy | | PubMed |  |  |
| Search formula | AND | (("RCT"[Title] OR "randomi*"[Title]) NOT ("protocol"[Title] OR "meta analysis"[Title] OR "review"[Title] OR "secondary analysis"[Title] OR "non randomi*"[Title])) |  |  |
|  |  | ("ipt"[Title/Abstract] OR "interpersonal therapy"[Title/Abstract] OR "interpersonal psychotherapy"[Title/Abstract] OR "interpersonal psychological"[Title/Abstract]) |  |  |
| Publication Years | | 01/2019 and 12/2023 |  |  |
| Languages | | English, German |  |  |
| Schema Therapy | | PubMed |  |  |
| Search formula | AND | (("RCT"[Title] OR "randomi*"[Title]) NOT ("protocol"[Title] OR "meta analysis"[Title] OR "review"[Title] OR "secondary analysis"[Title] OR "non randomi*"[Title])) |  |  |
|  |  | "schema"[Title/Abstract] |  |  |
| Publication Years | | 01/2019 and 12/2023 |  |  |
| Languages | | English, German |  |  |
| Dialectical Behavior Therapy | | PubMed |  |  |
| Search formula | AND | (("RCT"[Title] OR "randomi*"[Title]) NOT ("protocol"[Title] OR "meta analysis"[Title] OR "review"[Title] OR "secondary analysis"[Title] OR "non randomi*"[Title])) |  |  |
|  |  | ("dbt"[Title/Abstract] OR "dialecti*"[Title/Abstract]) |  |  |
| Publication Years | | 01/2019 and 12/2023 |  |  |
| Languages | | English, German |  |  |
| Cognitive Behavioral Analysis System of Psychotherapy | | PubMed |  |  |
| Search formula | AND | (("RCT"[Title] OR "randomi*"[Title]) NOT ("protocol"[Title] OR "meta analysis"[Title] OR "review"[Title] OR "secondary analysis"[Title] OR "non randomi*"[Title])) |  |  |
|  |  | ("Cognitive Behavioral Analysis System of Psychotherapy"[Title/Abstract] OR "CBASP"[Title/Abstract]) |  |  |
| Publication Years | | 01/2019 and 12/2023 |  |  |
| Languages | | English, German |  |  |
| Psychodynamic Therapy | | PubMed |  |  |
| Search formula | AND | (("RCT"[Title] OR "randomi*"[Title]) NOT ("protocol"[Title] OR "meta analysis"[Title] OR "review"[Title] OR "secondary analysis"[Title] OR "non randomi*"[Title])) |  |  |
|  |  | ("psychodynami*"[Title/Abstract] OR "stpp"[Title/Abstract] OR "psychoanaly*"[Title/Abstract] OR "transference"[Title/Abstract] OR "mentali*"[Title/Abstract] OR "supportive expressive"[Title/Abstract] OR "analytic therapy"[Title/Abstract] OR "analytic psychotherapy"[Title/Abstract] OR "analytic treatment"[Title/Abstract] OR "dynamic therapy"[Title/Abstract] OR "dynamic psychotherapy"[Title/Abstract] OR "dynamic treatment"[Title/Abstract] OR "dynamic intervention"[Title/Abstract]) |  |  |
| Publication Years | | 01/2019 and 12/2023 |  |  |
| Languages | | English, German |  |  |
| Client-centered Therapy | | | PubMed |  |
| Search formula | | AND | (("RCT"[Title] OR "randomi*"[Title]) NOT ("protocol"[Title] OR "meta analysis"[Title] OR "review"[Title] OR "secondary analysis"[Title] OR "non randomi*"[Title])) |  |
|  |  |  | ("process experiential"[Title/Abstract] OR "person center*"[Title/Abstract] OR "patient center*"[Title/Abstract] OR "client center*"[Title/Abstract] OR "non directive"[Title/Abstract] OR "Rogerian"[Title/Abstract] OR "humanistic"[Title/Abstract] OR "person focus*"[Title/Abstract] OR "client focus"[Title/Abstract] OR "patient focus*"[Title/Abstract] OR "person oriented"[Title/Abstract] OR "client oriented"[Title/Abstract] OR "patient oriented"[Title/Abstract]) |  |
| Publication Years | | | 01/2019 and 12/2023 |  |
| Languages | | | English, German |  |
| Systemic Therapy | | | | PubMed |
| Search formula | | | AND | (("RCT"[Title] OR "randomi*"[Title]) NOT ("protocol"[Title] OR "meta analysis"[Title] OR "review"[Title] OR "secondary analysis"[Title] OR "non randomi*"[Title])) |
|  |  |  |  | ("systemic therap*"[Title/Abstract] OR "system oriented"[Title/Abstract] OR "family therap*"[Title/Abstract] OR "family based"[Title/Abstract] OR "family oriented"[Title/Abstract] OR "systemic family therapy"[Title/Abstract] OR "Systemic Group Therapy"[Title/Abstract] OR "systemic approach*"[Title/Abstract] OR "systemic intervention*"[Title/Abstract] OR "systemic techni*"[Title/Abstract] OR "multisystemic"[Title/Abstract]) |
| Publication Years | | | | 01/2019 and 12/2023 |
| Languages | | | | English, German |
| Mindfulness-based Therapy | | | | PubMed |
| Search formula | | | AND | (("RCT"[Title] OR "randomi*"[Title]) NOT ("protocol"[Title] OR "meta analysis"[Title] OR "review"[Title] OR "secondary analysis"[Title] OR "non randomi*"[Title])) |
|  |  |  |  | ("mindful*"[Title/Abstract] OR "MBCT"[Title/Abstract] OR "MBSR"[Title/Abstract] OR "MBEAT"[Title/Abstract]) |
| Publication Years | | | | 01/2019 and 12/2023 |
| Languages | | | | English, German |
| Acceptance and Commitment Therapy | | | | PubMed |
| Search formula | | | AND | (("RCT"[Title] OR "randomi*"[Title]) NOT ("protocol"[Title] OR "meta analysis"[Title] OR "review"[Title] OR "secondary analysis"[Title] OR "non randomi*"[Title])) |
|  |  |  |  | ("psychological flexibility"[Title/Abstract] OR "act" [Title/Abstract] OR "acceptance and commitment" [Title/Abstract] OR "acceptance commitment" [Title/Abstract]) |
| Publication Years | | | | 01/2019 and 12/2023 |
| Languages | | | | English, German |
| Cognitive Behavioral Therapy | | | | PubMed |
| Search formula | | | AND | (("RCT"[Title] OR "randomi*"[Title]) NOT ("protocol"[Title] OR "meta analysis"[Title] OR "review"[Title] OR "secondary analysis"[Title] OR "non randomi*"[Title])) |
|  |  |  |  | ("CBT"[Title/Abstract] OR "GCBT"[Title/Abstract] OR "process based"[Title/Abstract] OR "trauma focus*"[Title/Abstract] OR "TF-CBT"[Title/Abstract] OR "cognitive based"[Title/Abstract] OR "cognitive therap*"[Title/Abstract] OR "cognitive treatment*" [Title/Abstract] OR "cognitive intervention*"[Title/Abstract] OR "cognitive behavio*"[Title/Abstract] OR "cognitive training" [Title/Abstract] OR "meta cognitive" [Title/Abstract] OR "metacognitive" [Title/Abstract] OR "cognitive restructur*"[Title/Abstract] OR "behavior activation"[Title/Abstract] OR "behaviour activation" [Title/Abstract] OR "behavior therap*"[Title/Abstract] OR "behaviour therap*"[Title/Abstract] OR "behavior treatment*" [Title/Abstract] OR "behaviour treatment*"[Title/Abstract] OR "behavior intervention*" [Title/Abstract] OR "behaviour intervention*"[Title/Abstract] OR "behavior training"[Title/Abstract] OR "behaviour training" [Title/Abstract] OR "exposure therap*"[Title/Abstract] OR "emotion regulation"[Title/Abstract] OR "emotional regulation" [Title/Abstract] OR "social skills training"[Title/Abstract] OR "social skills therapy"[Title/Abstract] OR "social skills treatment" [Title/Abstract] OR "social skills intervention"[Title/Abstract] OR "relaxation training"[Title/Abstract] OR "relaxation therapy" [Title/Abstract] OR "relaxation treatment"[Title/Abstract] OR "relaxation intervention" [Title/Abstract] OR "PMR" [Title/Abstract] OR "autogenic" [Title/Abstract] OR "progressive muscle relaxation" [Title/Abstract] OR "biofeedback"[Title/Abstract] OR "neurofeedback"[Title/Abstract]) |
| Publication Years | | | | 01/2019 and 12/2023 |
| Languages | | | | English, German |
| Internet- and Mobile-based Therapy | | | | PubMed |
| Search formula | | | AND | (("RCT"[Title] OR "randomi*"[Title]) NOT ("protocol"[Title] OR "meta analysis"[Title] OR "review"[Title] OR "secondary analysis"[Title] OR "non randomi*"[Title])) |
|  |  |  |  | ("eHealth"[Title/Abstract] OR "e-Health"[Title/Abstract] OR "electronic Health"[Title/Abstract] OR "mHealth"[Title/Abstract] OR "m-Health"[Title/Abstract] OR "mobile Health"[Title/Abstract] OR "e mental"[Title/Abstract] OR "e therapy"[Title/Abstract] OR "icbt" [Title/Abstract] OR "ccbt" [Title/Abstract] OR "computer assisted"[Title/Abstract] OR "online therap*"[Title/Abstract] OR "online treatment*"[Title/Abstract] OR "online intervention*"[Title/Abstract] OR "online training"[Title/Abstract] OR "online base*"[Title/Abstract] OR "online delivered"[Title/Abstract] OR "internet therap*"[Title/Abstract] OR "internet treatment*"[Title/Abstract] OR "internet intervention*" [Title/Abstract] OR "internet base*"[Title/Abstract] OR "internet delivered"[Title/Abstract] OR "digital therap*"[Title/Abstract] OR "digital intervention*"[Title/Abstract] OR "digital treatment*"[Title/Abstract] OR "therapy app"[Title/Abstract] OR "mobile intervention"[Title/Abstract] OR "mobile app*"[Title/Abstract] OR "mobile therapy"[Title/Abstract] OR "mobile treatment"[Title/Abstract] OR "web therap*"[Title/Abstract] OR "web based therap*"[Title/Abstract] OR "web based intervention*" [Title/Abstract] OR "smartphone" [Title/Abstract] OR "online self help"[Title/Abstract] OR "video therapy"[Title/Abstract] OR "virtual therapy"[Title/Abstract] OR "virtual reality"[Title/Abstract] OR "virtual intervention"[Title/Abstract] OR "self guided therapy"[Title/Abstract] OR "self help app"[Title/Abstract] OR "guided self help"[Title/Abstract] OR "blended care"[Title/Abstract] OR "blended treatment"[Title/Abstract] OR "blended therap*"[Title/Abstract] OR "blended intervention" [Title/Abstract] OR "digital mental" [Title/Abstract] OR "app based" [Title/Abstract] OR "telehealth" [Title/Abstract] OR "telemental health"[Title/Abstract] OR "remote therap*"[Title/Abstract] OR "remote care"[Title/Abstract] OR "gamified therap*"[Title/Abstract] OR "gamification"[Title/Abstract]) |
| Publication Years | | | | 01/2019 and 12/2023 |
| Languages | | | | English, German |
